# Simulating the impact of recombination rate on genomic selection breeding outcomes

**DOI:** 10.1093/g3journal/jkag049

**Published:** 2026-02-24

**Authors:** Zsa Zsa Boyny, Nicholas Lester, Karen Massel, Owen Powell, Rod J Snowdon, Sven E Weber

**Affiliations:** Department of Plant Breeding, Justus Liebig University Giessen, Giessen 35392, Hesse, Germany; Queensland Alliance for Agriculture and Food Innovation, The University of Queensland, Brisbane, QLD 4072, Australia; Queensland Alliance for Agriculture and Food Innovation, The University of Queensland, Brisbane, QLD 4072, Australia; Queensland Alliance for Agriculture and Food Innovation, The University of Queensland, Brisbane, QLD 4072, Australia; Department of Plant Breeding, Justus Liebig University Giessen, Giessen 35392, Hesse, Germany; Department of Plant Breeding, Justus Liebig University Giessen, Giessen 35392, Hesse, Germany

**Keywords:** quantitative genetics, plant breeding simulations, intrachromosomal recombination

## Abstract

Recombination shuffles alleles during meiosis, driving genetic diversity and shaping the outcomes of breeding programs. By breaking the physical links between loci, recombination facilitates the creation of new allelic combinations that can be selected for to improve genetic gain. Increasing the recombination rate by methods such as genome editing has become a goal for accelerating breeding. However, the effect of increased recombination rate on a population scale on breeding programs is not fully understood. We therefore carried out simulations to determine the effect of recombination on genetic gain in a breeding program using phenotypic and genomic selection, respectively. We focused on how heritability, number of quantitative trait loci, recombination rate increase factor, marker density, and training frequency affect breeding success. We also tested whether it is possible to use historic training sets without changes in recombination rate and merge the pre and postrecombination populations to improve prediction accuracy and genetic gain in genomic selection. We found that increasing recombination is particularly beneficial for highly quantitative traits with low heritability. However, with genomic selection, increasing recombination requires a higher training frequency as well as an increased marker density to accelerate superiority over phenotypic selection in terms of genetic gain. Furthermore, our simulations show that maintenance of old training sets and merging of training sets with different recombination rates is possible, but a decrease in prediction accuracy is expected, favoring frequent training and high marker density under increased recombination rates.

## Introduction

Breeding efforts rely on intrachromosomal recombination to break linkage and shuffle genetic diversity, enabling breeders to select advantageous alleles (Zou et al. 2024). As Muller (1964) and Felsenstein (1974) demonstrated, recombination is critical for population improvement. However, during meiosis crossovers (COs) are limited, requiring at least one obligate CO per chromosome but rarely exceeding 3 in most species (Mercier et al. 2015). Moreover, COs are predominantly confined to distal and subdistal euchromatic regions, with 80% occurring in approximately 25% of the genome (Blary and Jenczewski 2019; Brandariz and Bernardo 2019). This uneven distribution exacerbates linkage drag and hinders the introgression of beneficial traits, particularly for genes located in CO-poor regions (The International Barley Genome Sequencing Consortium 2012; Bauer et al. 2013; Choulet et al. 2014). Consequently, increasing recombination rates has great potential to accelerate the accumulation of advantageous alleles and the elimination of deleterious alleles during selection (Tourrette et al. 2019; Zou et al. 2024).

Historically, breeding programs could generally only increase recombination events by extending generational cycles, a time-intensive strategy that ultimately delays genetic progress (Bernardo 2009). Hence, alternative strategies to increase recombination were explored to overcome the time limitation. One approach involved inducing stress events during meiosis to influence the frequency of chiasmata and COs (Stern 1926; White 1934; Elliott 1955; Griffing and Langridge 1963; Modliszewski and Copenhaver 2017). Indeed, Verde et al. (2025) have proposed intentional drought stress as a method for increasing recombination in maize. Efforts were also made to identify natural variants with inherently higher recombination rates, with limited success, for example, in the model plant *Arabidopsis* and in barley (Ziolkowski et al. 2017; Dreissig et al. 2020). More recently, with the advancement of biotechnological tools, the ability to artificially increase recombination rates has emerged as a promising solution. Appropriate techniques have been reviewed extensively (Mercier et al. 2015; Choi 2017; Blary and Jenczewski 2019; Taagen et al. 2020; Zou et al. 2024).

Advancements in the understanding of genetic and epigenetic regulation of recombination, combined with genome editing tools, have led to novel approaches for increasing recombination rates. These include targeted double-strand breaks (eg CRISPR-Cas9), altering epigenetic marks, manipulating CO distribution by reversing the telomere-centromere gradient, and deleting chromosome regions (Hayut et al. 2017; Blary and Jenczewski 2019; Zou et al. 2024). Additionally, studies have identified key anti-CO factors—such as *FANCONI ANEMIA COMPLEMENTATION GROUP M* (*FANCM*), *RECQ HELICASE 4 (RECQ4)*, and *AAA-ATPase Fidgetin-like-1 (FIGL1)*—that suppress successful CO formation. Mutations in these genes significantly increase recombination frequency in *Arabidopsis*. For example, 3-fold increases in single *fancm* mutants, up to 6-fold increases in *recq4a/recq4b* mutants, and 2.5-fold increases in *figl1* mutants (Girard et al. 2015; Serra et al. 2018). These mutations can be combined for more extreme increases, with triple mutants of *RECQ4A*, *RECQ4B*, and *FIGL1* achieving up to a 7.8-fold increase in *Arabidopsis* (Fernandes et al. 2018). Overexpression of CO-promoting genes such as HEI10 has also been demonstrated to elevate recombination rate (Ziolkowski et al. 2017; Serra et al. 2018; Durand et al. 2022). However, manipulating recombination rates poses trade-offs, particularly regarding fertility, which is inconsistent across plant species (Mercier et al. 2015; Fernandes et al. 2018; Mieulet et al. 2018).

Given the resource-intensive nature of breeding programs, it is critical to assess the costs, benefits, and long-term impacts of increased recombination rates. Simulations have proven valuable in predicting genetic gain and optimizing breeding pipelines. McClosky and Tanksley (2013) simulated an 11% increase in genetic gain when recombination frequency was increased to the theoretical maximum of complete independence. Similarly, Battagin et al. (2016) reported genetic gains of up to 33.4% with a 20-fold increase in genetic map size, and these findings have been corroborated by subsequent studies (Gonen et al. 2017; Brandariz and Bernardo 2019; Ru and Bernardo 2019; Tourrette et al. 2019; Epstein et al. 2023).

These studies also revealed nuances regarding the effects of recombination. Tourrette et al. (2019) and Taagen et al. (2022) found that increased recombination is particularly advantageous when beneficial alleles are linked to undesirable alleles, but can be disadvantageous when beneficial alleles are tendentially linked together. Moreover, genomic selection (GS), a widely adopted tool in modern breeding, introduces additional considerations, as GS allows breeders to predict phenotypes using genomic markers and statistical models to overcome phenotyping limitations (Crossa et al. 2017). Increased recombination presents challenges for GS, including reduced prediction accuracy, particularly for traits with low heritability (Johnsson et al. 2022; Taagen et al. 2022). Battagin et al. (2016) also assumed the need for larger datasets under elevated recombination rates, a conclusion echoed by Tourrette et al. (2019) and Taagen et al. (2022), who emphasized the importance of denser marker sets and more frequent model training in the case of elevated recombination.

Despite these insights, the relationship between marker density, recombination rate, and accuracy of GS has not been fully elucidated. Nor is it clear how increased recombination would affect the long-term outcomes of breeding programs that utilize GS compared to conventional phenotypic selection. Additionally, the immediate impact of an altered recombination rate on prediction accuracy warrants further exploration, particularly regarding the usability of older training datasets for populations with different recombination rates. The feasibility of merging datasets originating from different recombination frequencies also requires investigation.

Here, we simulated these parameters using the important crop plant *Sorghum bicolor* (sorghum) as a genetic model. Sorghum is a C4 crop with a diploid genome (∼730 Mb) distributed across 10 chromosomes. In comparison to closely related species like maize, it generally shows superior heat and drought tolerance, traits which are becoming increasingly relevant in the face of climate change (Chadalavada et al. 2021). This study aims to analyze the impact of increased recombination on genetic gain, genetic variance, and prediction accuracy in both traditional phenotypic selection and GS frameworks. By addressing questions such as whether older training sets remain usable, how marker density requirements change, and how datasets from varying recombination frequencies can be combined, the study seeks to provide insights for optimizing breeding strategies under increased recombination conditions.

## Material and methods

### Experimental landscape

Simulations were performed in the RStudio environment (Version 4.0.0) using the package AlphaSimR (Version 1.6.1) (Gaynor et al. 2021). For the simulations, the *S. bicolor* reference genome version 3.1 (McClosky and Tanksley 2013) was utilized along with the single-nucleotide polymorphism (SNP) set of the Sorghum Genome Science Database (SorGSD Version 2.0, https://ngdc.cncb.ac.cn/sorgsd/). The SNP set comprises the reference genome positions of 33,825,236 SNP loci originating from 298 diverse accessions (Liu et al. 2021). Of these, 10,000 SNPs per chromosome were randomly sampled, and the base pair positions translated into centimorgan (cM) positions using a custom sorghum tool utilizing the consensus genetic map of Mace et al. (2009). The resulting map spanned 1545.1 cM along the 10 chromosomes of Sorghum (see Table 1). Based on the resulting map, the genome was generated in AlphaSimR. As a further parameter, the per base pair mutation rate was set to 3.0 × 10^−8^ to match the *Panicoideae* subfamily, similar to the analysis performed by Clark, Tavaré, and Doebley (2005).

**Table 1.** Genetic length in centimorgans of the 10 chromosomes of *Sorghum bicolor* under the normal recombination rate used for the simulations.

Chromosome	Genetic length
1	186.3
2	226.5
3	168.2
4	169.4
5	118.5
6	165.2
7	132.6
8	131.7
9	134.6
10	112.1

### Simulation parameters

For all different simulations, various parameters were tested. In all simulations, selection for a single trait influenced by either 10, 100, or 1,000 bi-allelic quantitative trait loci (QTL) per chromosome and with a narrow-sense heritability of either 0.2, 0.5, or 0.8 was simulated, to examine the respective influence of increased recombination on oligogenic to highly polygenic traits. QTL on each chromosome were randomly sampled from the 10,000 SNPs. Subsequently, QTL effects were sampled from a standard normal distribution and scaled to an additive genetic variance of $\sigma_{A}^{2}=1$ in the founder population. Only additive effects on traits were considered. Desired levels of narrow-sense heritability were achieved by sampling random errors from a standard normal distribution adequately scaled. Additionally, for the GS, simulations included SNP chips with different numbers of bi-allelic markers (between 100 and 1,000 per chromosome) by sampling 100, 200, 500, or 1,000 of the initial 10,000 SNPs, excluding QTL. Thereby, the SNPs were randomly sampled without filtering for minor allele frequency.

During the simulation, AlphaSimR automatically simulates genetic recombination according to the gamma model (McPeek and Speed 1995), using the genetic map to model the distribution and accounting for CO interference (Gaynor et al. 2021). To simulate an increased recombination rate, all positions on the genetic map were multiplied by the factor by which the recombination rate was increased. Thereby, 2-, 4-, and 8-fold increase were tested, representing the realizable range currently achievable using gene knockouts (Fernandes et al. 2018).

### Breeding scheme

**Fig. 1.**
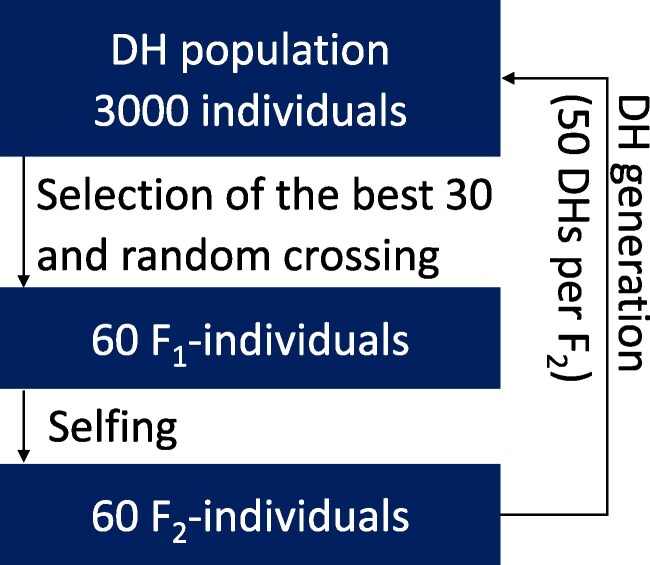
One cycle of simulated selection, selfing, and double haploid generation performed during the breeding program.

Based on the experimental landscape and simulation parameters, all simulations were initialized with a burn-in phase for 10 breeding cycles using phenotypic selection for the trait to remove initial bias. After this burn-in, linkage disequilibrium (LD) levels, genetic structure, and genetic diversity were intended to resemble those of a population that had already undergone a recent, realistic breeding program prior to any modification of recombination parameters, consistent with observations reported by Bančič et al. (2024). For this, a founder population of 3,000 homozygous genotypes was simulated as described above. In this base population, the top 30 individuals with the highest performance of the simulated trait were selected based on the phenotype from which 60 random crosses were generated. Subsequently, 1 F_2_-individual was generated from each F_1_-individual by selfing. Finally, a double haploid (DH) population with 50 individuals was generated from each F_2_-individual, leading to 3,000 simulated DH lines on which the next selection was performed (Fig. 1). During the breeding program no new genetic variation was introduced. After 10 breeding cycles of phenotypic selection, selfing, and DH generations, different simulations were performed as described below. For comparability, the genetic gain was set to 0 and genetic variance was scaled to 1 after the burn-in phase. To account for stochastic differences, all simulations were replicated 100 times and the averages over those runs were taken for comparison.

### Simulating the long-term influence of recombination rate changes

To investigate the long-term effects of altered recombination rates, simulations were conducted over 15 additional breeding cycles after burn-in with both normal and increased recombination rates. Two scenarios were considered. In the first scenario, phenotypic selection, as implemented during the burn-in phase, was maintained over the 15 breeding cycles under both normal and increased recombination conditions. In the second scenario, GS was employed. For each DH population, a ridge regression best linear unbiased prediction (RRBLUP) model was trained using the phenotype and genotype data of the whole current generation. The genomic estimated breeding values (GEBVs) from this model were used to select 30 individuals from the same DH population that were subsequently used for simulated crosses. To evaluate the effect of model training frequency, additional simulations were performed where the model was trained every second and fourth cycle instead of every cycle. During each simulation cycle, several metrics were measured, including the population average genetic value, average genetic variance, and average genic variance. Thereby, the genic variance is the sum of the expected variance under Hardy–Weinberg- and linkage equilibrium at all QTL, and the genetic variance is the variance observed in the population. Using these metrics, the Bulmer effect, the deviance of additive-genetic variance from disequilibrium (Bulmer 1971) was calculated following the formula provided by Taagen et al. (2022), dividing the genetic variance by the genic variance divided by 2, to account for the fully inbred state of the DHs. Additionally, the genetic gain per genetic variance was calculated to compare how effectively genetic variance was translated into genetic gain. To compare the genetic improvement under increased recombination scenarios with the normal recombination scenario, the gain ratio was calculated by dividing the genetic value in the 15th cycle of each increased recombination scenario by the genetic value from the normal recombination scenario. When GS was applied, prediction accuracy was assessed in each generation using the Pearson correlation coefficient between the estimated breeding values and the actual genetic values. Additionally, the variability between simulation runs for each scenario was analyzed by calculating the variance of the average genetic gain across 100 replications.

### Maintenance of training sets across recombination rate changes

In a second simulation, the effect of using models trained on different recombination rates was examined. After the burn-in phase, an RRBLUP model was trained, and selection was performed based on the GEBVs. The recombination rate was then either maintained or altered, followed by 1 cycle of crossing, selfing, and DH production, as previously described. The previously trained model was subsequently applied to the resulting generation, and prediction accuracy was calculated. Accuracy was recorded both in the generation used for training and in the generation where the model was reapplied. To examine the applicability, the change in prediction accuracy was quantified by subtracting the accuracy of the second generation from that of the generation in which the model was generated.

### Using merged training-sets with different recombination rates

Finally, to study the opportunity to merge datasets with different recombination rates, the final 3 DH generations during the burn-in process were merged and used as a training set to predict GEBV and select in the last DH population of the burn-in process. Subsequently, the recombination rate was altered, and the genotypes of the next DH generated were added to the training set while the oldest genotypes were removed. Within the new cycle selection was performed based on the predicted GEBV. Consequently, the steps of selection, crossing, selfing, and DH generation were repeated for 3 more breeding cycles. Thereby, the model was retrained every cycle using the current and the previous 2 generations for training. As a benchmark, the same process was performed with no change in recombination rate. In each generation, the population mean genetic value, the genetic and genic variance, and prediction accuracy were calculated. Based on the results of 2.1 and 2.2, this simulation was only performed for 100 QTL per chromosome, a heritability of 0.5 and a 4-fold recombination rate increase.

## Results

In our simulations, we focused on the question of how increasing the recombination rate influences the breeding framework. We concentrated on the difference in the influence of increased recombination rates on phenotypic selection vs GS. In these scenarios, the need for increased marker sets as well as the training frequency, the opportunity to maintain training sets from before the recombination rate change, and merging of training populations with different recombination rates were studied. Furthermore, the influence of the number of QTL, heritability, and the degree of recombination rate increase was also tested for both phenotypic selection and GS.

In all simulations similar trends were observable. Thus, if not stated otherwise the results for the scenario with 100 QTL per chromosome, a heritability of 0.5, a recombination rate increase of 4, and 500 markers per chromosome for the genotyping during the GS are displayed.

### Phenotypic selection with increased recombination

**Fig. 2.**
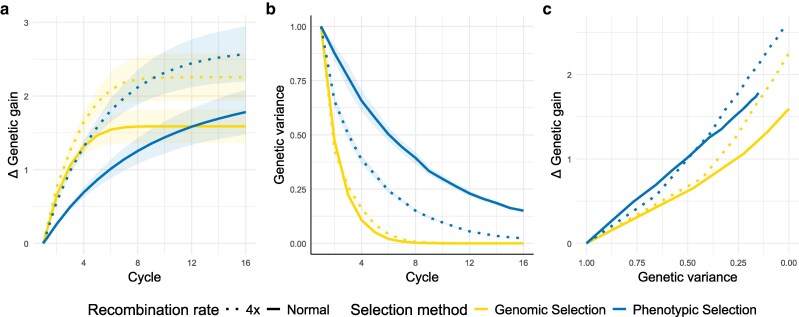
Mean of Δ genetic gain (a) and genetic variance (b) per cycle as well as the genetic gain per genetic variance lost (c) over 100 replications. Solid lines show genetic gain per genetic variance under normal crossover rate; dotted lines show the effect of a 4-fold increase in crossover rate. Blue plots indicate phenotypic selection, while yellow plots indicate genomic selection using 500 markers per chromosome and training every generation. The ribbons represent the variance between the replicates. The trait is influenced by 100 markers per chromosome and has a heritability of 0.5.

The results for simulations of phenotypic selection with increased recombination demonstrate that increasing the intrachromosomal recombination rate enhances both the magnitude and speed of genetic gain through phenotypic selection. This effect becomes evident from the very first cycle after the increase in recombination rate. Especially in the first generation, an increase in the recombination rate accelerates the genetic gain (Fig. 2a and Supplementary Fig. A in File 1). Specifically, scenarios with an 8-fold increase in recombination achieve the highest genetic gain, on average 2.79 after 15 breeding cycles. This is followed by 4- and 2-fold increases, with gains of 2.57 and 2.33, respectively, compared to 1.78 under normal recombination. With higher recombination rates, genetic variance is more effectively translated into genetic gain. In terms of gain ratios, the 8-fold increase yields 1.57, while the 4- and 2-fold increases result in ratios of 1.44 and 1.31, respectively (for a comprehensive overview, see Supplementary Table A in File 1).

When studying the different number of QTL influencing a trait, more QTL led to a slower, however, eventually higher increase of the genetic gain and a higher gain ratio after 15 breeding cycles. We found that lower heritability led to a slower increase in genetic gain during the first breeding cycles. However, after 15 breeding cycles, it resulted in higher overall gain and a slower depletion of genetic variance. Consistently, the gain ratio was slightly higher after 15 breeding cycles under lower heritability.

Increased recombination rate resulted in higher variability between replicates, particularly after 15 breeding cycles. Notably, across the 100 simulations, the scenarios involving an 8-fold increase resulted in genetic gain with a variance of 0.45, while the standard recombination rate resulted in a variance of just 0.22. The course of genetic variance over the 15 breeding cycles demonstrated that all scenarios with an increased recombination rate led to a faster decline in variance compared to normal recombination (Fig. 2b). This indicates that with phenotypic selection, a higher intrachromosomal recombination rate accelerates the reduction of genetic and genic variance. However, this corresponds to a faster and higher genetic gain, indicating a more efficient conversion of variance into gain. An 8-fold increase in CO rate sustained genetic variance for a slightly longer duration than a 4-fold increase, which in turn persisted for a longer period than a 2-fold increase. Regarding the translation of genetic variance into genetic gain, an increased recombination rate results in a more efficient translation of variance into gain when looking in later breeding cycles. This trend changes with a decrease in the genetic variance (Fig. 2c, for further details see Supplementary Fig. C in File 1).

The Bulmer effect quantifies the loss of genetic variance due to negative covariance between loci under selection. In the context of standard recombination, the Bulmer effect demonstrated relative stability over a span of 15 breeding cycles in most scenarios, exhibiting only minor increases, contingent upon the number of QTL influencing the trait and heritability. Conversely, under conditions of increased recombination, an initial decline in the Bulmer effect was observed, subsequently followed by a gradual rise. The more precise selection allowed a stronger selection for favorable alleles, causing an initial drop followed by an increase of the Bulmer effect due to a steady and effective selection. The extent of this discrepancy in outcomes varied according to the trait architecture, with scenarios featuring a higher number of QTL and lower heritability demonstrating a trend more akin to that observed under normal recombination, while those featuring fewer QTL and higher heritability exhibited a more pronounced deviation from this trend (for further details see Supplementary Fig. D in File 1).

### Genomic selection with increased recombination

GS with training in each generation showed comparable trends for genetic gain to those seen for phenotypic selection. Higher CO rates led to higher genetic gain (Fig. 2a). On average, a gain of 2.37 was reached by cycle 15 in the scenarios with an 8-fold increase in recombination, 2.25 for a 4-fold increase, and 2.37 for a 2-fold increase. In contrast, a gain of just 1.59 was achieved in scenarios with a normal recombination rate. This translates to gain ratios of 1.49, 1.42, and 1.31 for 8-, 4-, and 2-fold increase in CO rate (for a comprehensive overview see Supplementary Table 1). As in the simulations using phenotypic selection, an increase in recombination rate resulted in a higher variance between the replications (Fig. 2a). The increased genetic gain is reflected in the prediction accuracy, with increased recombination rates leading uniformly to a higher prediction accuracy. A prediction accuracy of 0.82 was observed in the first cycle under normal recombination, while an average accuracy of 0.94 was observed for the increase factors under scenarios with a higher recombination rate in the first cycle after the burn-in. In all simulations, the prediction accuracy subsequently dropped due to exhaustion of the genetic variance and the fixation of alleles in the population. For all simulations, we found that a higher marker density enabled a less severe decline in prediction accuracy in the following generations. This effect was especially noticeable with increased recombination rates. In most scenarios, the accuracy was generally maintained at levels above the scenarios with a normal recombination rate (for further details see Supplementary Fig. E in File 1). To further study the interplay between marker density and recombination rates, it would be interesting to also study the linkage decay under different recombination landscapes in real populations.

While phenotypic selection outperformed GS in the long run, GS improved genetic gain faster in the short term, and this trend was maintained under increased recombination rates. However, phenotypic selection under increased recombination achieved almost the same short-term gain as GS under normal recombination. In contrast to phenotypic selection, GS was able to maintain variance for slightly longer under scenarios with elevated recombination rates (Fig. 2b and Supplementary Fig. B in File 1). Hereby, we observed that the higher the increase, the longer the preservation of genetic variance was sustained. In the context of GS, the Bulmer effect exhibits a more pronounced and rapid trend compared to phenotypic selection. Under normal recombination rates, the Bulmer effect initially declines due to the introduction of negative covariance among alleles, decreasing the genetic variance while decreasing the genic variance to a lesser extent. In later generations, the effect rises, approaching infinity around cycle 5 in our simulations, due to a stronger decrease of genic variance than of genetic variance. Increasing the recombination rate delays this divergence and amplifies the magnitude of the initial decline by faster separating superior from inferior alleles (see Supplementary Fig. D in File 1). In contrast to phenotypic selection in the scenarios with GS, the variance is always translated better into genetic gain (Fig. 2c, for further details see Supplementary Fig. C in File 1).

In GS, genetic gain increased with marker density especially with many QTL influencing the trait. However, under both normal and increased recombination, a saturation effect was visible. Consequently, increasing density from 100 to 200 markers per chromosome led to a larger gain increase than an increase from 500 to 1,000 markers per chromosome. With increased CO rates, differences attributable to marker density increased, and the saturation of genetic gain required higher marker density. This trend is especially pronounced in highly quantitative traits. Furthermore, in terms of the number of QTL influencing the trait as well as heritability, the trends were congruent with the observations under phenotypic selection. A higher number of QTL influencing the trait led to a higher genetic gain and higher gain ratio, while a lower heritability resulted in a higher gain after 15 breeding cycles (for further details see Supplementary Fig. A in File 1).

Reducing the frequency of model training resulted in lower total gain over the 15 simulated breeding cycles, especially in the first breeding cycles and with low heritability and a high number of QTL. The absolute gain after 15 breeding cycles decreased in most scenarios with less frequent training. When training was performed only every second generation, the absolute gain decreased to 2.13, and when training was performed every fourth generation, it decreased further to 2.07. In terms of gain ratio, training every cycle resulted in a ratio of 1.42, while training every second and every fourth generation resulted in a reduced ratio of 1.37. This trend was especially pronounced when many QTL influenced the trait, heritability was low, and recombination was increased 4- or 8-fold. In contrast, when only 10 QTL per chromosome were simulated or heritability was high, less frequent model retraining did not consistently perform worse than training in every cycle (for further details see Supplementary Table A and Fig. F in File 1).

### Evaluating cross-generational model training having different recombination rate

**Fig. 3.**
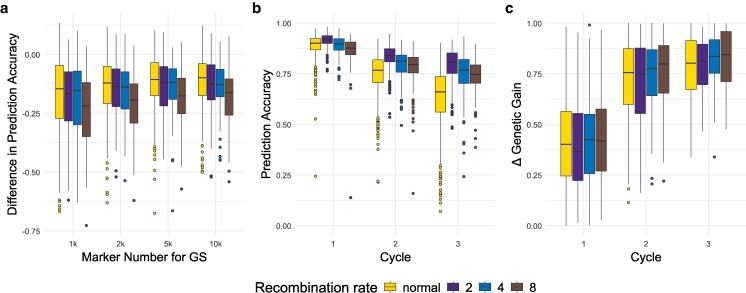
(a) Change of prediction accuracy in relation to the accuracy of the previous generation. The model was trained in the previous generation and applied in both. Prediction accuracy (b) and genetic gain (c) in the 3 generations after the burn-in phase. The model was trained on the generation and the previous 2 generations. In scenarios with a change in CO rate the rate is increased between breeding cycles 0 and 1. For all figures, the trait was influenced by 100 QTL per chromosome with a heritability of 0.5. A total of 500 markers per chromosome were used for the genomic prediction.

GS models remained accurate even when the training population had a different recombination rate than the testing population. However, the prediction accuracy decreased to a greater extent when reusing the model on the next cycle generated under conditions with increased recombination rates. The strongest decline in accuracy was observed under an 8-fold increase in CO rate (Fig. 3a, Supplementary Fig. G in File 1). Reduced numbers of markers, an increased number of QTL, and lower heritability caused a more severe decline in prediction accuracy.

### Impact of recombination rate on model performance

With training sets containing merged phenotypic and genotypic data from multiple generations with different recombination rates, we observed that higher marker densities of 500 or 1,000 markers per chromosome consistently resulted in higher prediction accuracy across generations and more stable accuracy under increased recombination rates. In all simulations, the prediction accuracy declined over the breeding cycles due to a decline of genetic variance as well as the breakage of the linkage between causal variance and SNPs. However, by having more markers the accuracy was maintained for longer. Furthermore, increasing the recombination rate led to a slower reduction in prediction accuracy, with the smallest decrease seen with a 2-fold increase in CO rate (Fig. 3b and Supplementary Fig. H in File 1).

Genetic gain did not clearly differ between the different recombination rates in the simulated breeding cycles; however, a slight superiority was observed in the last breeding cycles for scenarios with increased recombination rate (Fig. 3c and Supplementary Fig. H in File 1). Furthermore, scenarios with increased recombination maintained genetic variance for more generations when using 3 generations to train the model.

Similarly, an increased number of markers generally led to a slower decline of prediction accuracy, especially under increased recombination rate scenarios, as well as a higher overall genetic gain. Scenarios with increased recombination rates performed slightly higher in this regard. Again, trends in regard to the number of QTL and heritability held true in this simulation.

## Discussion

Using breeding program simulations based on the sorghum genome, we showed that increasing the recombination rate can be advantageous for both phenotypic selection and GS breeding strategies. However, increasing the recombination rate demands an increased marker set and more frequent training when utilizing GS. Furthermore, we studied the opportunities and challenges when applying and merging datasets with different recombination rates when using GS. While it was possible to maintain and include datasets from different recombination rates, we found that it is advantageous to use models trained on the same generation to avoid an accelerated decline in prediction accuracy due to the disruption of linkage between markers and QTL.

### Enhanced recombination rate increases genetic gain, however, variance does not react uniformly

Over a 15-breeding cycle study of various scenarios, higher recombination rates consistently led to increased genetic gain, regardless of the number of QTL, heritability, or selection method. The positive influence can be explained in part using the breeder's equation: $\Delta G=ir_{a}\sigma_{G}$. Where *i* is the selection intensity, $r_{a}$ the selection accuracy, and $\sigma_{G}$ the genetic variance. While the breeder's equation describes the efficiency of selection based on genetic variation and selection accuracy, higher recombination facilitates the creation of new allele combinations, which releases novel genetic variance during meiosis in each generation that can be exploited by selection. This observation is consistent with other simulations, for example, by Battagin et al. (2016) or McClosky and Tanksley (2013), who demonstrated that increasing the CO rate enhances genetic gain by promoting the independent assortment of alleles, thereby facilitating selection.

We found that the magnitude of the positive effect of increased recombination is dependent on the genetic architecture of the trait as well as the selection methods. For traits governed by many QTL, increased recombination is especially beneficial. This was also described by Tourrette et al. (2019), who emphasized that enhanced recombination can alleviate the effects of tight linkage between QTL, a crucial factor influencing the outcome of breeding programs. Their work aligns with our findings by demonstrating that the effect of increased recombination scales with trait complexity, as seen in traits governed by numerous QTL. For traits influenced by only a small number of QTL, genetic drift and the low number of QTL needing to be reshuffled lead to a limited success of increased recombination. In addition, when total heritability is held constant, reducing the number of QTL increases the average genetic variance per QTL, which facilitates selection.

Our simulations further showed that increasing the recombination rate is more beneficial for traits with lower heritability. Under low heritability, genetic progress is slower, which allows increased recombination to uncover more beneficial genetic combinations over time. In contrast, under high heritability, the trait is already largely optimized through efficient selection, making the additional benefit from increased recombination less significant. Furthermore, it can be expected that if increasing the recombination increases the genetic variance ($\sigma_{G}$), with negligible effects on the residual variance ($\sigma_{e}$), we can consequently expect an increase in trait heritability leading to a more precise selection and an acceleration of genetic gain. Bernardo (2009), McClosky and Tanksley (2013), and Tourrette et al. (2019) all found that different heritability only has a negligible effect on the gain ratio when increasing the recombination rate. Bernardo (2009) also noted that trends in simulations were reliant on the selection intensity, which may also have affected the simulations in our study.

We also show how recombination influences the maintenance of genetic variance under different selection methods. Increased recombination helps maintain genetic variance by counteracting genetic drift and enabling the independent selection of beneficial alleles, a trend that was previously observed by Tourrette et al. (2019) and Taagen et al. (2020, 2022). Overall, phenotypic selection was able to maintain genetic variance for longer than GS due to its imprecise sampling of alleles and slower fixation rates. For phenotypic selection, an increase in recombination rate enables a more precise selection of the ideal genotype and thereby faster depletion of the genetic variance. With an increase in recombination rate, phenotypic selection shows increased precision of selection and performs almost as well as GS. Under increased recombination, phenotypic selection shows reduced efficiency in converting genetic variance into gain during the first breeding cycles. In later breeding cycles, phenotypic selection translates variance into gain more effectively. Even with this reduced early translation efficiency, genetic gain still increases much faster under increased recombination, particularly during the initial breeding cycles.

Due to a longer variance retention, phenotypic selection outperformed GS in terms of long-term genetic gain across all scenarios by better translating genetic variance into genetic gain. However, it is important to note that our simulations assumed a single trait and did not account for the introduction of new genetic diversity, as would be expected in real-world breeding programs. When multiple traits are considered and genetic diversity is frequently introduced, GS would likely maintain its superiority for longer due to its higher accuracy and efficiency in identifying favorable alleles. On the other hand, this observation underlines the importance of repeated introduction of novel diversity to maintain higher genetic gains achieved by GS. However, depending on the origin of this new genetic material, the genetic gain could decrease afterward due to the introduction of noise and unfavorable alleles in addition to favorable alleles, as well as the divergence between material.

One notable consequence of increased recombination is the introduction of higher stochasticity due to the random distribution of COs along chromosomes. While this randomness increases the diversity of allele combinations, it can also occasionally disrupt beneficial linkages, particularly in populations with low initial diversity (Taagen et al. 2022). Thus, the net impact of recombination on genetic gain is context-dependent, influenced by both population structure and trait architecture. This point was previously discussed in detail, for example, by Taagen et al. (2022), who claimed that positive alleles tend to be coupled with each other, leading to a loss of genetic gain when increasing the recombination rate. However, correcting for this situation necessitates detailed knowledge of QTL distribution, which is not achievable for QTL in LD with one another. Furthermore, Bernardo (2017), Gonen et al. (2017), and Ru and Bernardo (2019) simulated the shift of recombination hotspot centers, thereby accessing genetic variance coupled with favorable traits, while also necessitating a detailed knowledge about QTL and their effects, which might be masked by LD.

These findings illustrate that increased recombination can amplify the effectiveness of both GS and phenotypic selection by enhancing the use of genetic variability. However, the outcomes of these selection methods diverge due to their intrinsic mechanisms. Although phenotypic selection benefits from broader alleles sampling and better but slower translation from variance into gain than GS, GS leverages precision and short-term efficiency, which might be more important for breeders Notably, the benefits of recombination are not universal; scenarios with coupled beneficial alleles may experience diminished or even adverse effects from higher recombination rates (Tourrette et al. 2021; Taagen et al. 2022).

### Marker density and training strategies: key factors for prediction accuracy

The relationship between marker density and prediction accuracy is crucial for optimizing GS models. Although increasing marker density enhanced predictive accuracy in our simulations, a saturation effect was observable. Under higher recombination rates, the saturation shifts, necessitate denser marker sets to maintain accuracy. This was already expected by Battagin et al. (2016) and Tourrette et al. (2019) and highlights the importance of marker density in counteracting the erosion of LD between markers and QTL.

Frequent recalibration of predictive models emerged as another critical factor for maintaining accuracy under elevated recombination rates, especially when having a high number of QTL influencing the trait and low heritability. The simulations demonstrated that recalibrating models every breeding cycle preserved higher accuracy compared to recalibrations every second or fourth generation. Conversely, infrequent recalibration diminished the benefits of increased recombination, contributing to stagnation in genetic gain. This effect was more pronounced in scenarios with low marker densities, underscoring the need to optimize recalibration frequency alongside marker density. Tourrette et al. (2019) provided similar evidence for the training frequency, illustrating the compounded negative effects of sparse recalibration and insufficient marker sets on GS efficacy. This effect might be especially pronounced in breeding schemes that rely on rapid cycling. For example, 2-step breeding strategies (Gaynor et al. 2017) might experience elevated genetic drift between population improvement and product development with increased recombination rates.

The complex interplay between marker density, recombination, and recalibration frequency underscores the necessity for integrated GS strategies. As stated before, it would be interesting to study the linkage decay under different recombination rates in real populations. Successful breeding programs must combine reasonably large marker sets with frequent recalibration to ensure sustained genetic progress, especially under elevated recombination frequencies. This is further illustrated by the fact that, in some simulations, use of GS and low marker density with infrequent training was inferior to scenarios relying on phenotypic selection from year 1 under increased recombination. This shows that phenotypic selection might be a feasible solution for a scenario with a limited budget to increase marker density or training frequency. This is particularly relevant for underutilized crops like sorghum for which funding for large breeding programs might be limited, but the benefits of increased recombination rates are still desired.

### Challenges and opportunities in integrating datasets with different recombination rates

We evaluated the transferability of genomic prediction models across populations with varying recombination rates, focusing on how changes in recombination rates, QTL characteristics, and marker densities influence prediction accuracy. These factors are critical for improving GS strategies and ensuring robust model performance over multiple generations with an increased recombination rate.

Our analysis revealed that increased recombination rates following model training reduced prediction accuracy when the model was reapplied. This decline results from the disruption of LD and the breakdown of QTL-marker associations, both essential for maintaining prediction accuracy. This effect was confirmed by the trend toward more severe decline in prediction accuracy with higher recombination rates. Marker density also emerged as a crucial determinant of prediction performance, especially for highly quantitative traits. Higher marker densities mitigated the adverse effects of increased recombination by preserving strong marker-QTL associations.

Using previously trained models generally reduced prediction accuracy. To address the applicability of older training sets, we tested whether merging populations during periods of changing recombination rates could enhance model performance. By retraining models across multiple generations using a rolling window approach—incorporating 3 consecutive training populations—we assessed the effect on prediction accuracy and potential for genetic gain. We observed that when merging multiple generations, an increase in recombination rate did not immediately lead to an increase in genetic gain, in contrast to scenarios with training on only 1 generation. This suggests that data from populations with varying recombination rates can disrupt precise selection, thereby limiting genetic gain.

Interestingly, the slowest decline in prediction accuracy occurred when recombination rates were increased by 2-fold, while more extreme increases led to sharper declines. This could reflect an equilibrium between the effects of recombination on breaking linkages among markers and QTL, while also taking advantage of the positive impact of increased recombination on prediction accuracy. Excessive recombination may excessively disrupt marker and QTL combinations when merging datasets from different breeding cycles, accelerating the breakdown of beneficial linkage among adjacent loci, whereas moderate increases in recombination promote genetic diversity without excessively fragmenting advantageous genetic structures. This is leading to a balance at a 2-fold increase with a better maintenance of prediction accuracy. Similar trends were observed when analyzing the effects of recombination rates on favorable and unfavorable allele pairings (Tourrette et al. 2019; Taagen et al. 2022). However, since this was not elaborated in our study, it is more likely that the duplicated training set closely resembled the set with normal recombination rates, benefiting from the inclusion of data from multiple generations as well as an increase in recombination rate.

De Roos et al. (2009) demonstrated that merging populations can enhance prediction accuracy, but this approach requires a dense marker set to counteract the effects of population divergence. Our simulations reaffirmed this finding, highlighting the importance of marker density in preserving prediction accuracy. Higher marker density not only improved prediction performance but also buffered the disruptive effects of CO events, particularly when numerous QTL influenced the trait. However, saturation was visible, hence there is a limit to the beneficial effect on prediction.

Nevertheless, our simulations demonstrate that it is possible to maintain and apply models trained on different populations or to merge data from populations with varying recombination rates. However, increased recombination rates are likely to reduce prediction accuracy and hinder genetic gain. Therefore, when feasible, it is advantageous to use models based on data from populations with similar recombination rates to preserve accuracy and maximize genetic progress.

### Potential and limitations of increasing the recombination rate

The results of our study underscore the significant potential that increased recombination holds for enhancing breeding program designs and advancing future research. By amplifying recombination rates, it becomes feasible to improve genetic gain, especially for traits governed by numerous QTL. These traits typically pose challenges in conventional breeding schemes due to the complex genetic architecture and the limited efficiency of selection. Enhanced recombination facilitates the disruption of LD between positive and negative variants in repulsion, thereby enabling more effective selection and improved capture of beneficial alleles.

However, increased recombination comes with risks. While breaking LD is advantageous for genetic gain, it may also disrupt beneficial co-adapted gene complexes (Tourrette et al. 2019; Taagen et al. 2022). It is also worth noting that artificially increasing recombination rates may not accurately reflect the true recombination landscape; empirical evidence suggests that such mutations often enhance recombination in regions that are already highly recombinant, rather than generating new breakpoints. This likely differs from how recombination is modeled in simulations (Fernandes et al. 2018). Using real recombination landscapes as Tourrette et al. (2019) and Taagen et al. (2022) for the normal recombination rate can increase the closeness to reality, however is still limited to the quality of the map. A further improvement could be achieved by using realistic landscapes of recombination rate increases, which are currently not available for Sorghum.

Beyond standard breeding efforts, increased recombination can play a transformative role in prebreeding programs. The approach can expedite the introgression of novel traits, ensuring higher diversification and enrichment with exotic alleles (Blary and Janczewski 2019). Enhanced recombination can also facilitate fine mapping of traits by increasing resolution, which is essential for pinpointing genes associated with desirable characteristics and breaking linkages in regions where CO events are rare. This can, in turn, enhance the efficiency of backcrossing, streamlining the process of transferring beneficial alleles into elite breeding lines, as shown in simulations by Tourrette et al. (2021).

As in previous simulation studies, our simulations assume fixed heritability, which oversimplifies breeding schemes and limits the ability to replicate the full complexity of a real-world breeding scenario. Similar limitations have been reported in other simulation-based studies (Epstein et al. 2023), necessitating implications to verify our results. Additionally, for the SNP-chips the SNPs were not filtered for minor allele frequency, which might have decreased the prediction accuracy but with a minimal impact for GBLUP models, as described by Zhang et al. (2019). Furthermore, by assuming the generation of DHs in a sorghum breeding program, where inbreds are generally used rather than DHs, the simulated breeding program does not perfectly depict realistic settings. Those additional generations of selfing would add more recombination even accelerating the predicted effect of increased recombination. Nevertheless, the observed trends can be assumed to be true for breeding programs with different outlines.

The practical realization of recombination control presents additional challenges. Biotechnological techniques such as CRISPR-based modification of recombination rates have shown promise but face barriers due to cost, labor intensity, legality in some countries, and technical challenges working in crop species (Fernandes et al. 2018; Blary and Jenczewski 2019). Research into biotechnological modulation of recombination remains mostly confined to model organisms rather than crop species. Long-term stability and potential trade-offs also need to be considered directly in crop species, which tend to have larger and more complex genomes than model systems. For instance, increased recombination has been associated with reductions in fertility and disruption of essential gene linkages, as observed in experimental studies on CO frequency manipulation (Mieulet et al. 2018). As recombination-increasing modifications may be undesirable in cultivars, methods to remove them at the end of a breeding cycle also need to be investigated, for example, maintaining them in heterozygous form during breeding (Zhang et al. 2017; Mieulet et al. 2018; Osman et al. 2024). Additionally, the sustainability of artificially induced recombination rates remains uncertain, necessitating further research into their stability over generations (Blary and Jenczewski 2019).

Despite these challenges, the exploration of increased recombination represents a promising frontier in plant breeding. By addressing existing limitations through continued research and technological advancement, breeders may unlock novel pathways to accelerate genetic improvement and develop more resilient, productive agricultural systems.

## Conclusion

The simulations presented here demonstrate that increasing the recombination rate is a powerful tool when using phenotypic selection and GS breeding frameworks and is especially valuable for the improvement of highly quantitative traits with low heritability. Increased recombination has the strongest effects in GS systems when combined with frequent retraining, high recombination, and dense marker coverage. Although trained models were transferable across populations with different recombination rates, and populations with different recombination rates could be merged, our simulations showed that retraining models under increased recombination improves prediction accuracy. Our simulations thereby present an important step toward the implementation of methods to increase the recombination rate in practical breeding programs.

## Supplementary Material

jkag049_Supplementary_Data

## Data Availability

All the scripts for reproducing the breeding scheme simulations performed are publicly available at 10.5281/zenodo.17063587. SNP data used can be found at SorGSD (https://ngdc.cncb.ac.cn/sorgsd/). Supplemental material available at G3 online.
