# Supplementary material for: Simulating the impact of recombination rate on genomic selection breeding outcomes
Source: G3 (Bethesda). 2026 Feb 24;16(5):jkag049. doi: 10.1093/g3journal/jkag049 (PMC13148392; doi:10.1093/g3journal/jkag049)
Supplement: jkag049_Supplementary_Data [file jkag049_supplementary_data.pdf]

Supplementary material

Table A:

Gain ratio, calculated by dividing the genetic gain in the 15th cycle of the scenario with the increased recombination by the scenario with the normal recombination for the different numbers of QTLs per chromosome(nQTL), Heritabilities (h^2), recombination rate increase factors (INC), marker number per chromosome and frequency of training the model used for genomic prediction.

Training	INC	Marker number	h^2 / nQTL	0.2: 10	0.2: 100	0.2: 1000	0.5: 10	0.5: 100	0.5: 1000	0.8: 10	0.8: 100	0.8: 1000
Every cycle	2	Pheno		1,26	1,42	1,49	1,24	1,31	1,48	1,23	1,25	1,33
		100		1,20	1,32	1,32	1,23	1,24	1,34	1,15	1,20	1,15
		200		1,18	1,31	1,36	1,20	1,28	1,41	1,15	1,22	1,24
		500		1,17	1,33	1,41	1,22	1,29	1,44	1,15	1,25	1,31
		1000		1,19	1,34	1,40	1,20	1,29	1,49	1,18	1,24	1,35
	4	Pheno		1,34	1,51	1,58	1,27	1,44	1,57	1,24	1,32	1,47
		100		1,23	1,37	1,38	1,19	1,37	1,34	1,17	1,26	1,21
		200		1,26	1,36	1,41	1,20	1,37	1,42	1,20	1,29	1,30
		500		1,22	1,36	1,46	1,20	1,42	1,49	1,15	1,32	1,42
		1000		1,24	1,36	1,46	1,17	1,42	1,51	1,16	1,33	1,51
	8	Pheno		1,33	1,58	1,74	1,41	1,57	1,77	1,21	1,40	1,78
		100		1,22	1,36	1,42	1,27	1,41	1,45	1,20	1,32	1,41
		200		1,24	1,38	1,47	1,29	1,47	1,48	1,16	1,35	1,54
		500		1,23	1,39	1,50	1,27	1,49	1,64	1,19	1,42	1,62
		1000		1,23	1,41	1,59	1,24	1,46	1,67	1,22	1,40	1,73
Every second cycle	2	100		1,23	1,28	1,29	1,25	1,27	1,21	1,23	1,11	1,25
		200		1,22	1,33	1,33	1,21	1,30	1,32	1,24	1,15	1,32
		500		1,23	1,35	1,36	1,25	1,35	1,37	1,23	1,16	1,38
		1000		1,19	1,32	1,42	1,24	1,36	1,37	1,26	1,19	1,44
	4	100		1,20	1,34	1,34	1,30	1,26	1,30	1,09	1,25	1,14
		200		1,20	1,41	1,43	1,27	1,31	1,42	1,12	1,29	1,24
		500		1,21	1,44	1,44	1,27	1,37	1,49	1,11	1,34	1,35
		1000		1,18	1,39	1,47	1,26	1,38	1,53	1,14	1,34	1,40
	8	100		1,30	1,33	1,38	1,19	1,34	1,33	1,25	1,25	1,23
		200		1,28	1,40	1,46	1,20	1,40	1,42	1,29	1,30	1,35
		500		1,26	1,39	1,50	1,18	1,44	1,53	1,26	1,38	1,49
		1000		1,28	1,38	1,51	1,18	1,47	1,57	1,27	1,37	1,56

Every fourth cycle	2	100	1,25	1,33	1,27	1,31	1,20	1,26	1,30	1,15	1,11
		200	1,25	1,37	1,39	1,27	1,23	1,29	1,28	1,16	1,22
		500	1,25	1,38	1,40	1,29	1,28	1,37	1,29	1,18	1,28
		1000	1,25	1,38	1,42	1,31	1,31	1,43	1,30	1,19	1,29
	4	100	1,26	1,34	1,36	1,23	1,29	1,30	1,20	1,22	1,20
		200	1,30	1,39	1,42	1,21	1,32	1,37	1,21	1,25	1,25
		500	1,27	1,40	1,48	1,19	1,34	1,46	1,25	1,26	1,39
		1000	1,26	1,39	1,49	1,20	1,39	1,52	1,26	1,32	1,46
	8	100	1,18	1,29	1,26	1,26	1,30	1,28	1,30	1,25	1,09
		200	1,27	1,36	1,36	1,29	1,33	1,37	1,23	1,29	1,27
		500	1,22	1,39	1,43	1,31	1,43	1,52	1,24	1,38	1,41
		1000	1,24	1,40	1,51	1,31	1,45	1,56	1,32	1,44	1,49

Figure A:

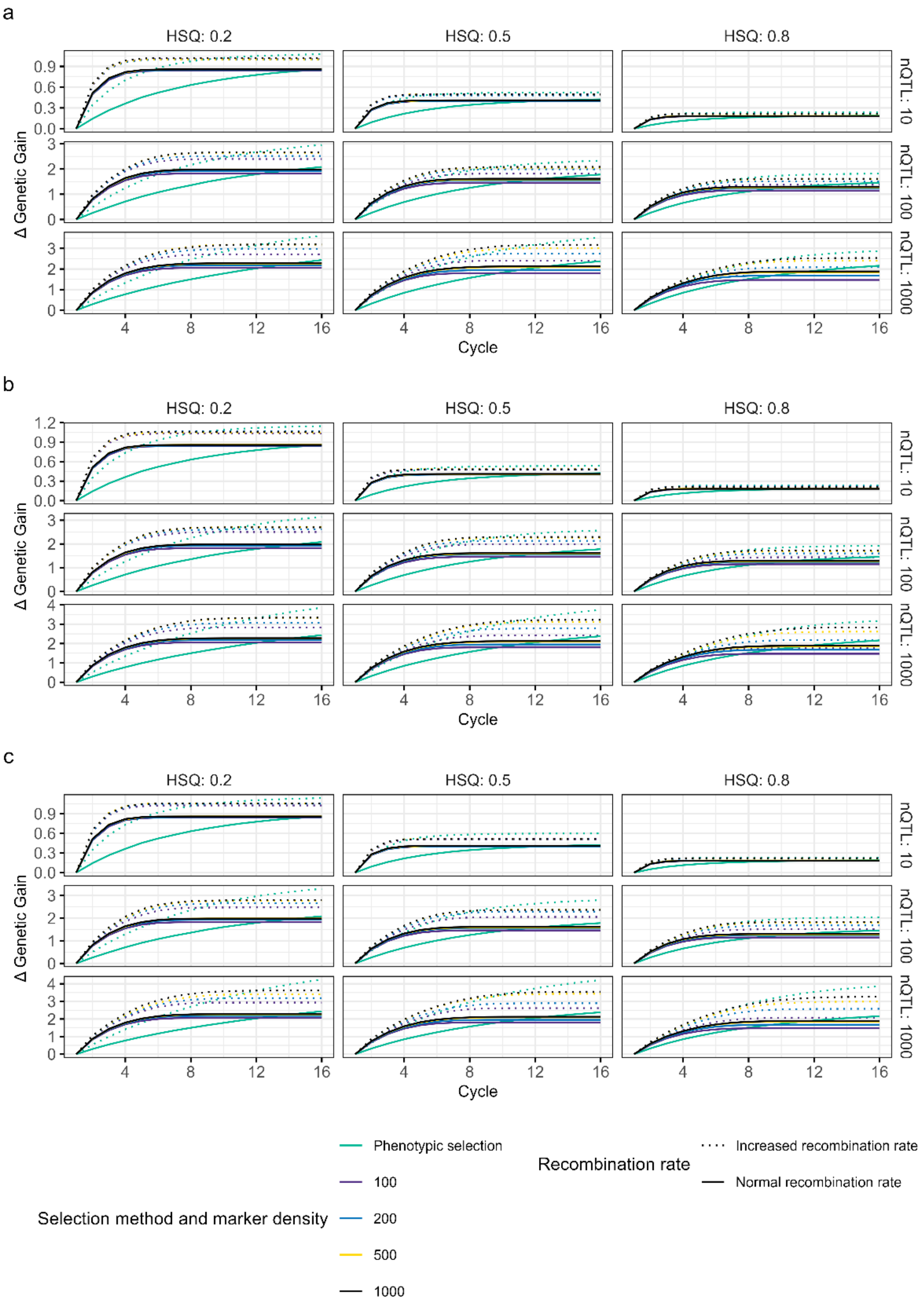

Genetic gain for traits having different heritabilities (HSQ) and numbers of QTL (nQTL) using genomic selection with different numbers of marker and phenotypic selection (green). For normal and 2- (a), 4- (b) and 8-times (c) increased recombination rate.

Figure B:

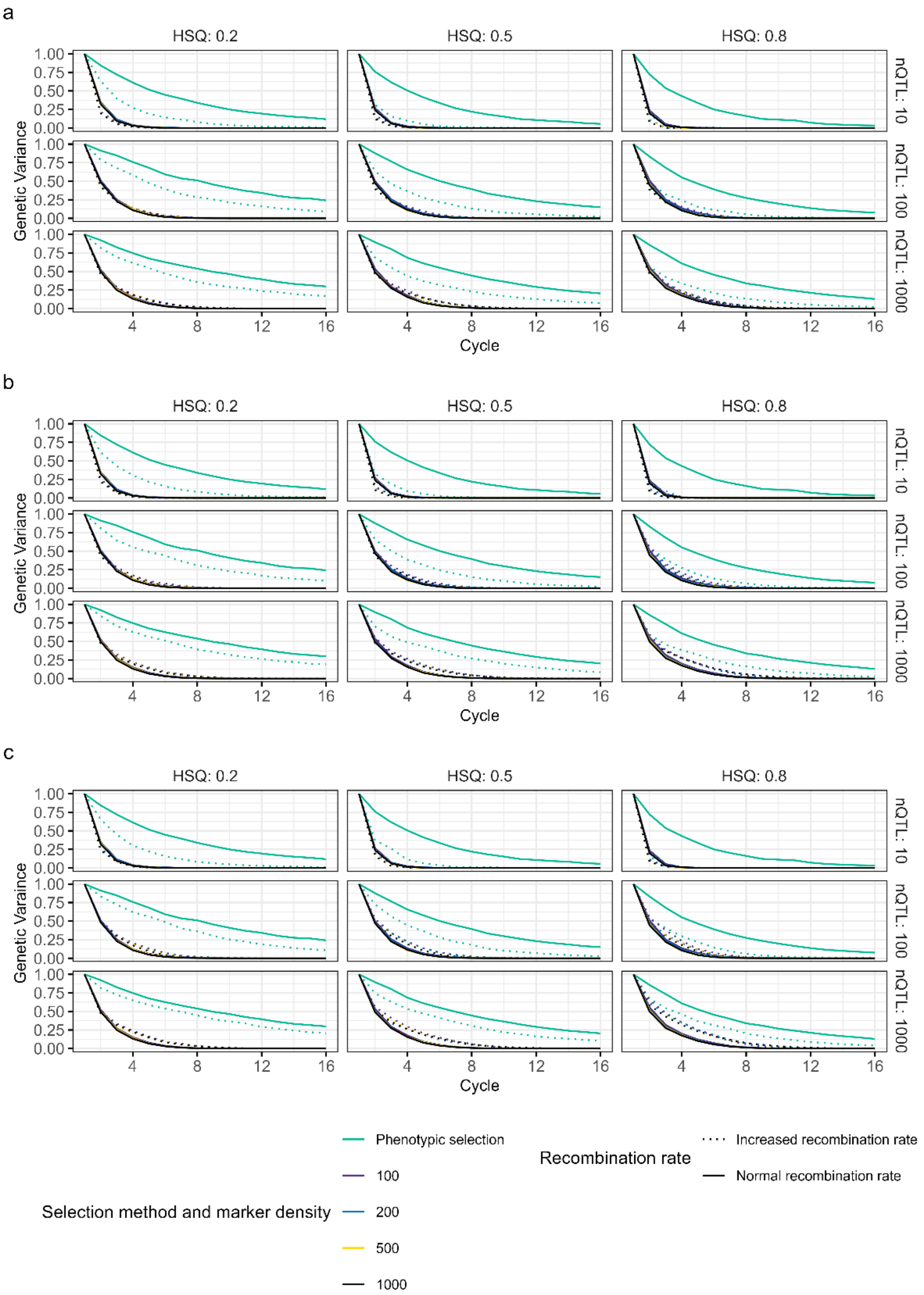

Genetic variance for traits having different heritabilities (HSQ) and numbers of QTL (nQTL) using genomic selection with different numbers of marker and phenotypic selection (green). For normal and 2- (a), 4- (b) and 8-times (c) increased recombination rate.

Figure C:

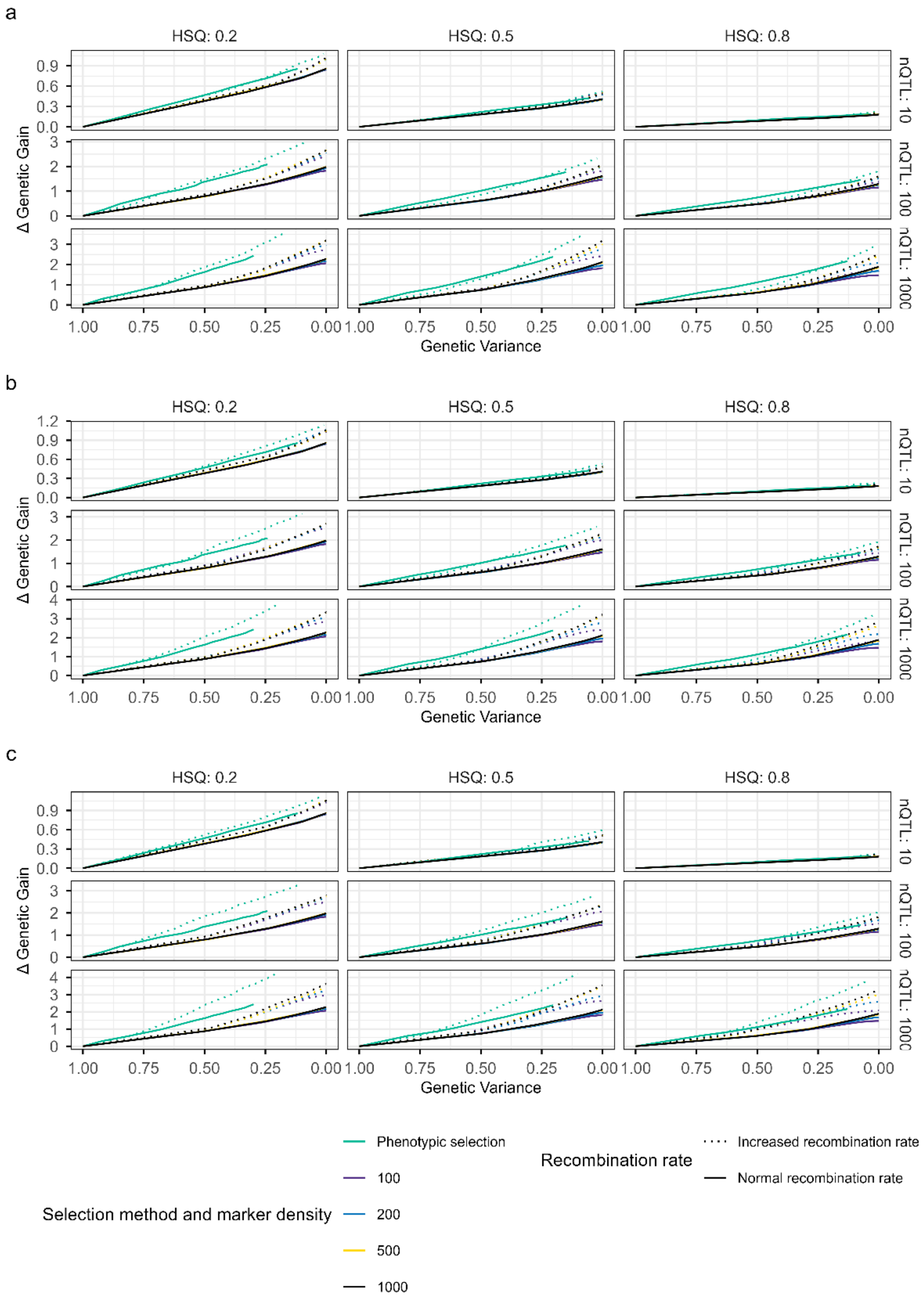

Genetic variance per genetic gain for traits having different heritabilities (HSQ) and numbers of QTL (nQTL) using genomic selection with different numbers of marker and phenotypic selection (green). For normal and 2- (a), 4- (b) and 8-times (c) increased recombination rate

Figure D:

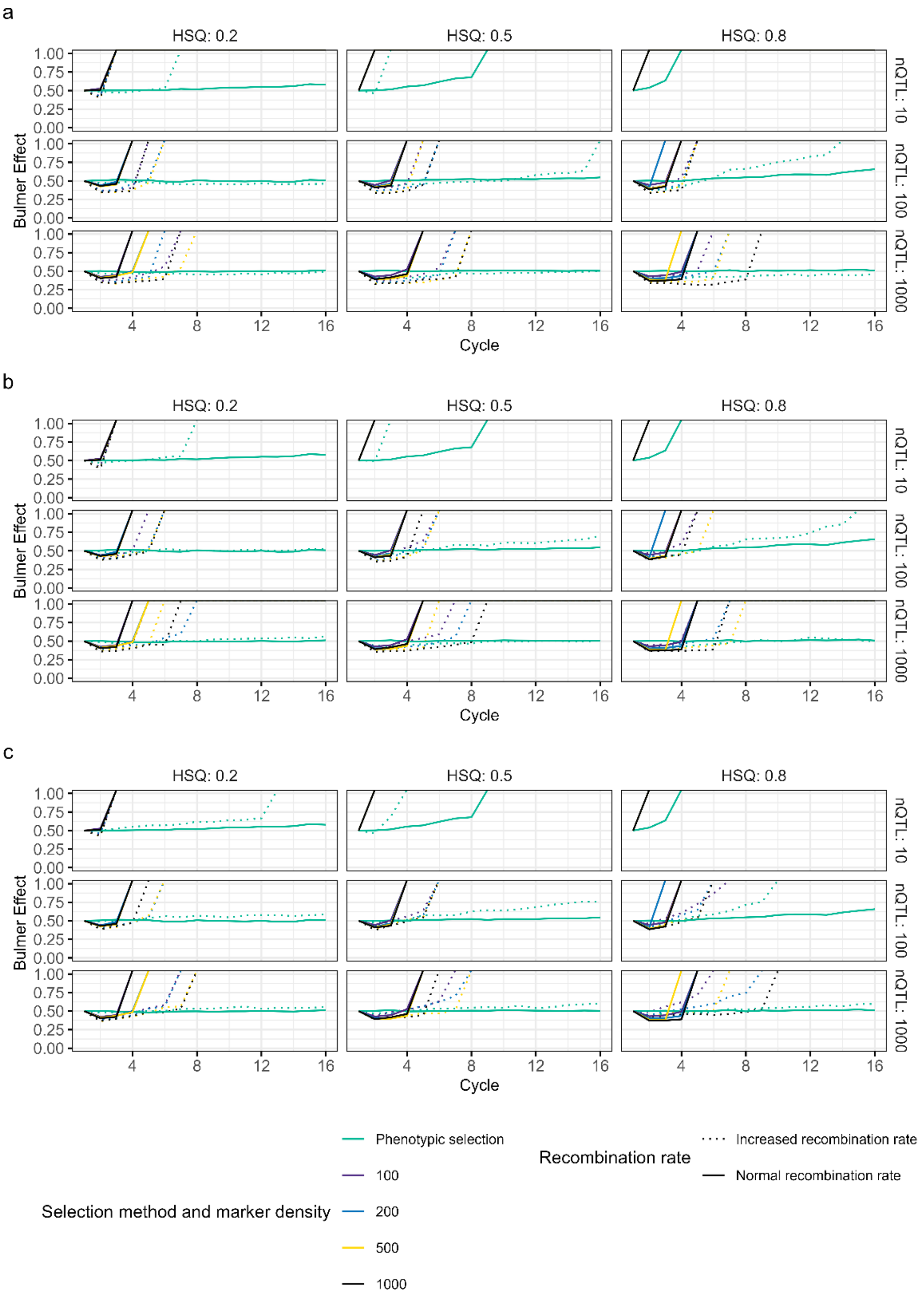

Bulmers effect for traits having different heritabilities (HSQ) and numbers of QTL (nQTL) using genomic selection with different numbers of marker and phenotypic selection (green). For normal and 2- (a), 4- (b) and 8-times (c) increased recombination rate. The lines that are cut show a Bulmer effect going to infinity, due to a total depletion of the genetic variance

Figure E:

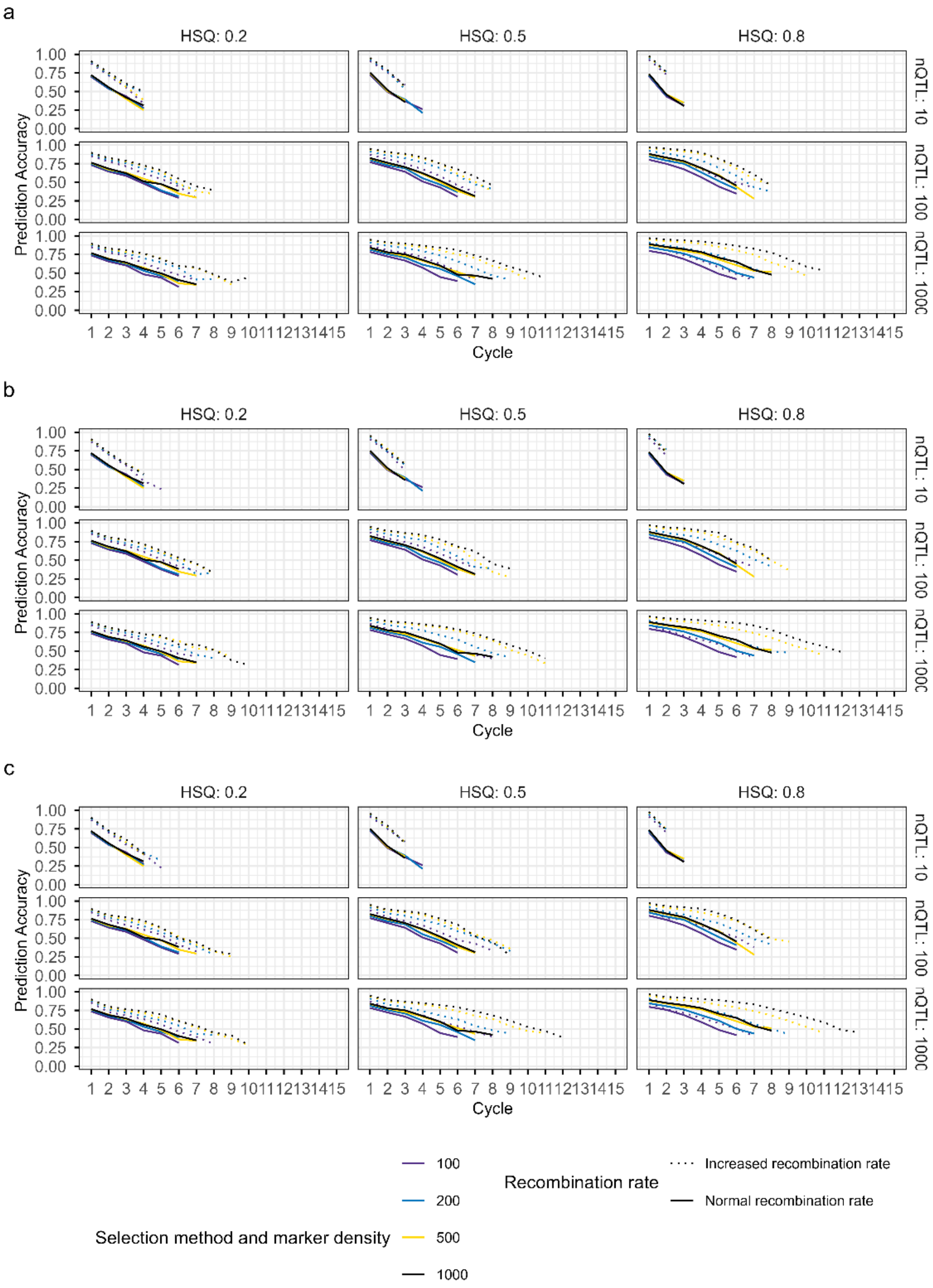

Prediction accuracy for traits having different heritabilities (HSQ) and numbers of QTL per chromosome (nQTL) using genomic selection with different numbers of marker. For normal and 2- (a), 4- (b) and 8-times (c) increased recombination rate. The lines that are cut show that the prediction accuracy is not anymore calculatable, due to a total depletion of the genetic variance

Figure F:

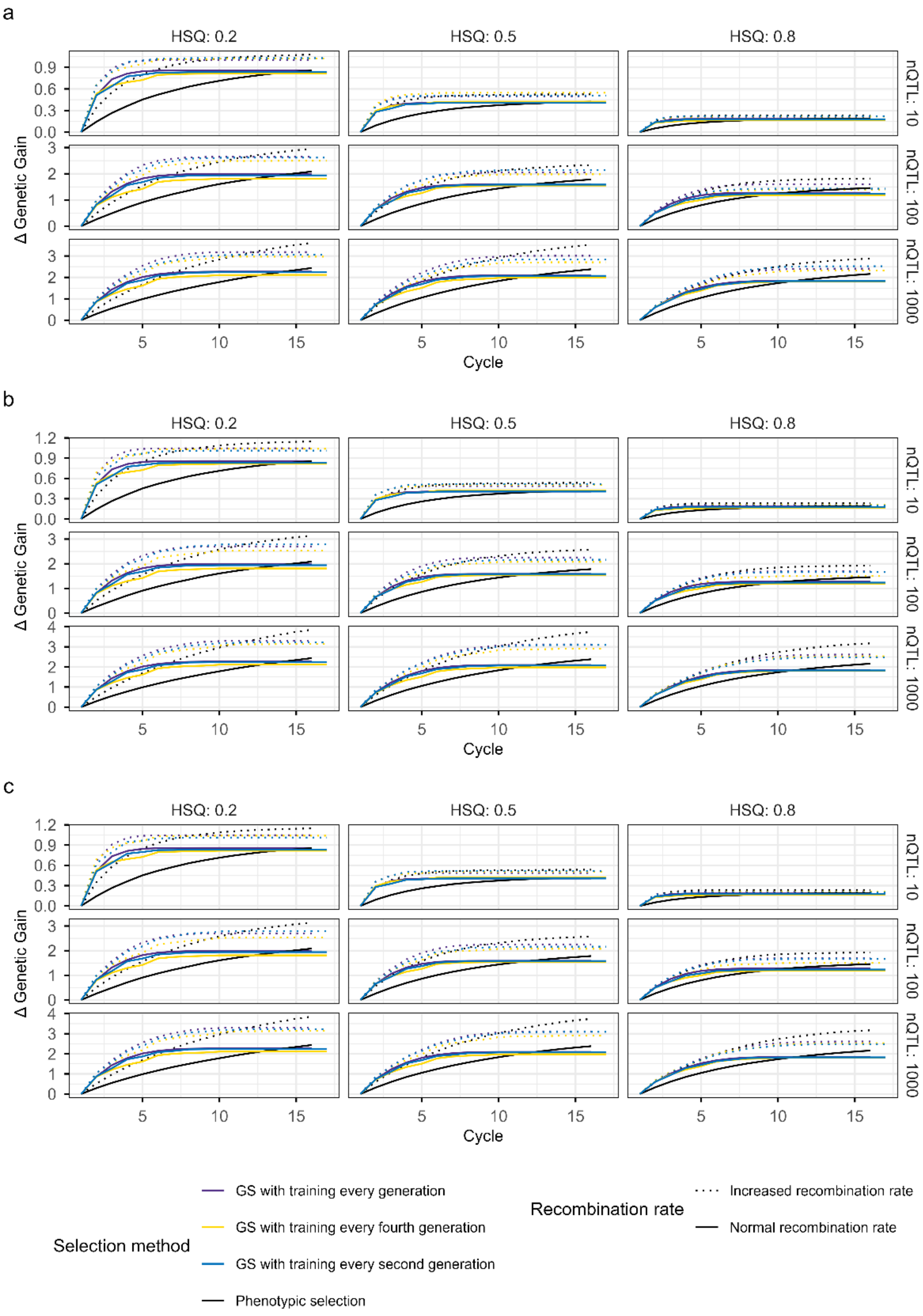

Genetic gain for traits having different heritabilities (HSQ) and numbers of QTL per chromosome (nQTL) using genomic selection with 500 markers and different training frequencies and phenotypic selection (green). For normal and 2- (a), 4- (b) and 8-times (c) increased recombination rate.

Figure G:

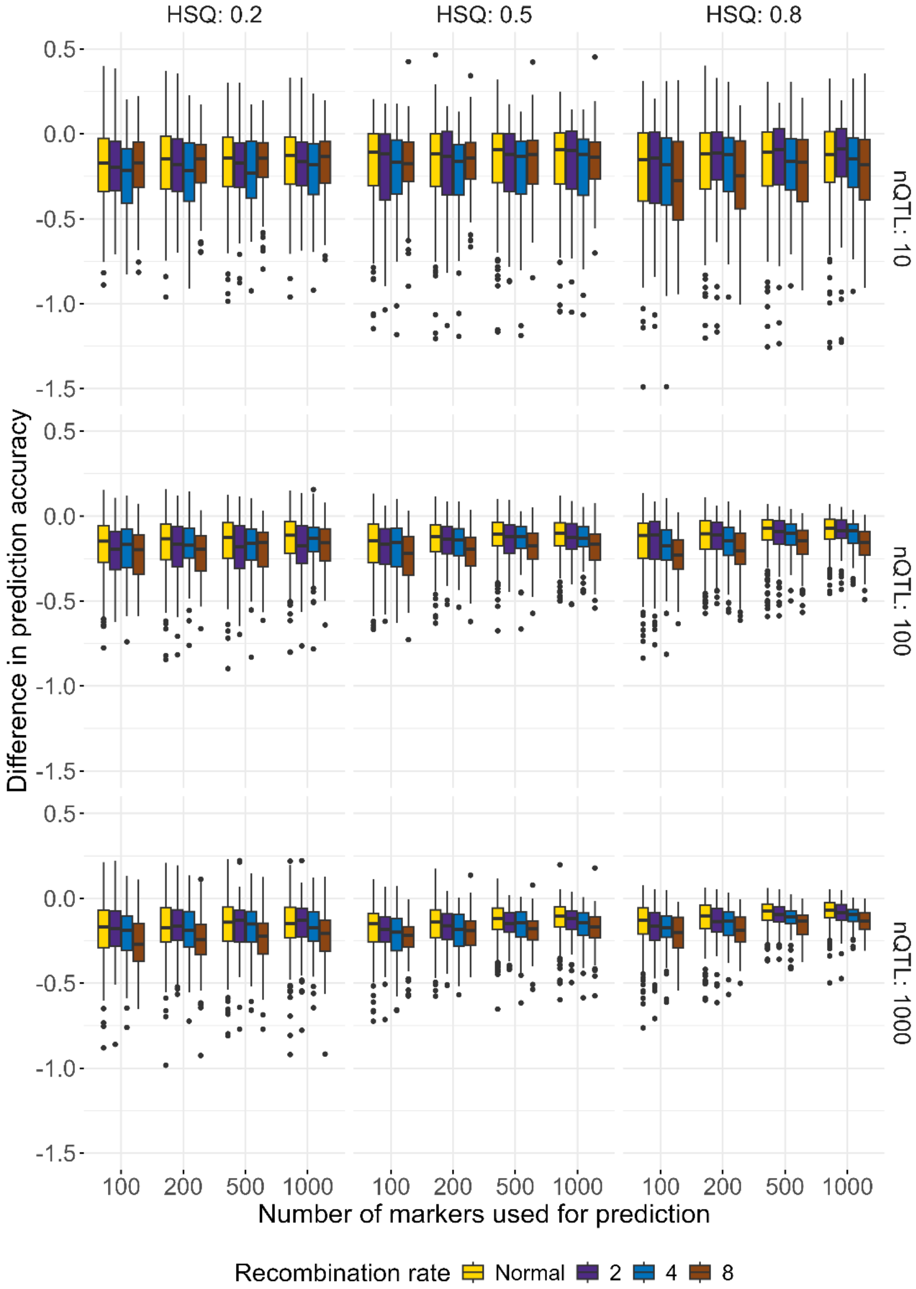

Change of prediction accuracy, calculated by subtracting the prediction accuracy before the generation rate change, in which the model was trained, of the population after the generation rate change and the increase of the recombination rate. Depicted for the different number of QTL (nQTL), heritabilities (HSQ) and marker densities used per chromosome, as well as for no recombination rate change (yellow), a duplication (purple), a fourfold (blue) and eightfold (brown) increase.

Figure H:

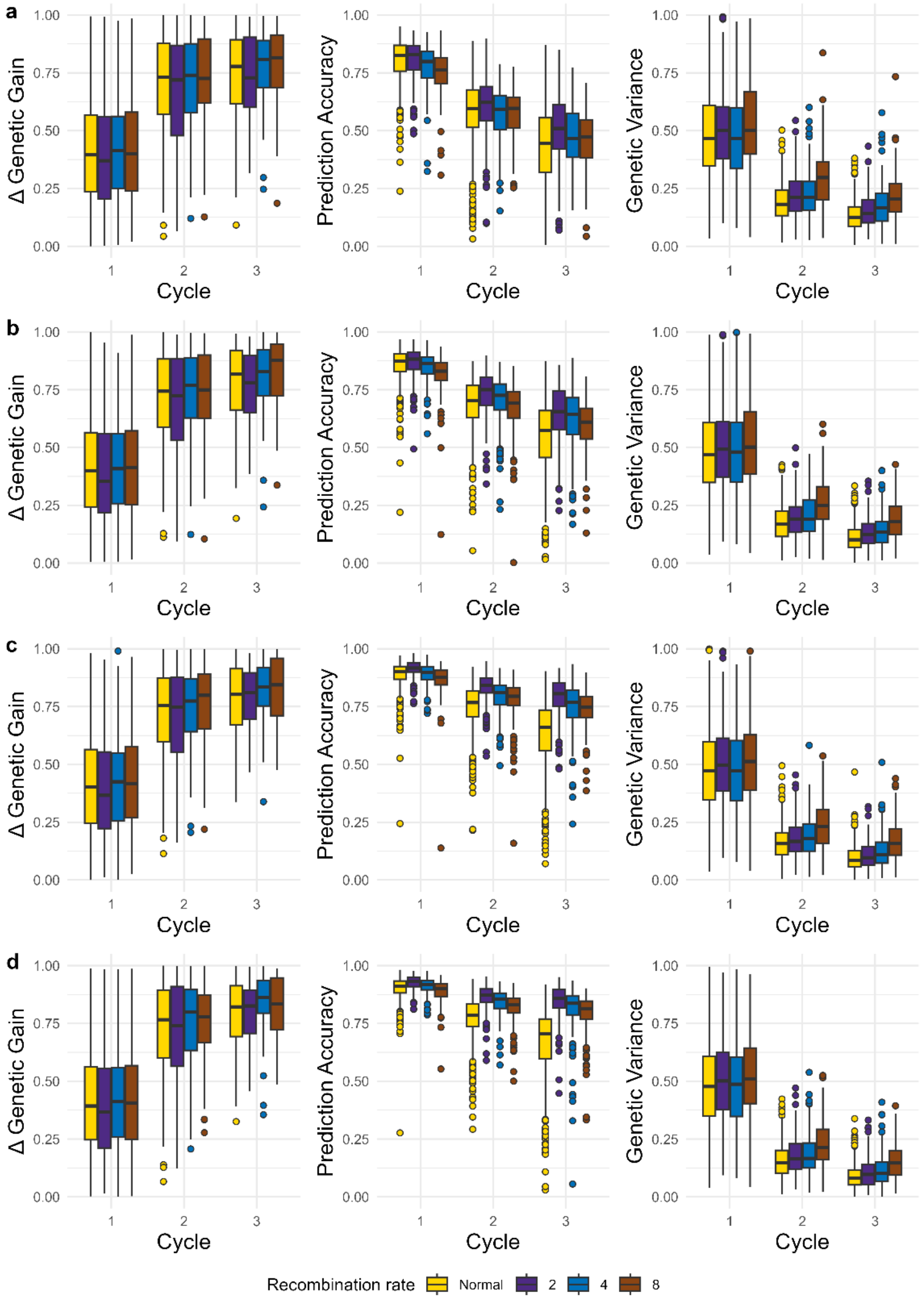

Genetic gain, Prediction accuracy and genetic variance in the three generations after the burn-in phase. Predictions are being made based on a RRBLUP model trained with the generation and the two previous ones. If changed the recombination rate is increased between cycle 0 and cycle 1. The trait is influenced by 100 QTLs per Chromosome with a heritability of 0.5. For the prediction 100 (a), 200 (b), 500 (c) and 1000 (d) Markers are used per chromosome.
